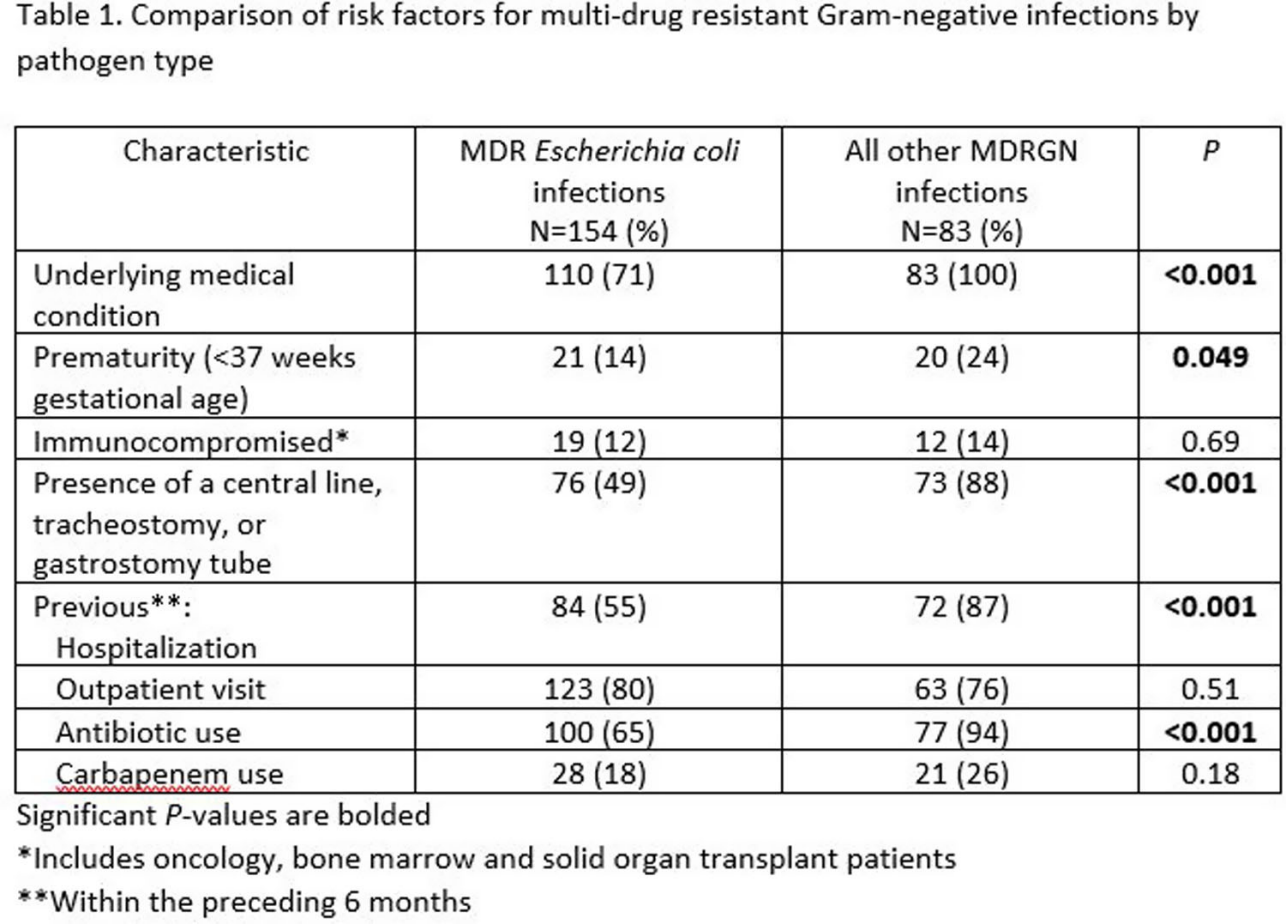# Risk Factors for Multi-Drug Resistant Gram-negative Infections across a Pediatric Hospital System

**DOI:** 10.1017/ash.2024.262

**Published:** 2024-09-16

**Authors:** Aarika Young, Elizabeth Tocco, Tjin Koy, Grant Stimes, Judith Campbell, Lucy Marquez, Catherine Foster

**Affiliations:** Texas Children’s Hospital; Baylor College of Medicine and Texas Children’s Hospital

## Abstract

**Background:** Infections due to antibiotic resistant bacteria are increasing worldwide and while, the epidemiology of these pathogens is well described in adults, pediatric specific data are lacking. We sought to gain an understanding of the risk factors for multi-drug resistant Gram-negative (MDRGN) infections in our pediatric population. **Methods:** We performed a retrospective review of pediatric patients seen at a pediatric hospital system in 2022 who had a culture-positive MDRGN, which was defined as a gram-negative bacteria resistant or intermediate to at least 1 antibiotic in ≥ 3 antibiotic groups. Repeat positive cultures for the same MDRGN were considered a single infection episode if occurring within a 14-day period. Demographic, clinical, and microbiologic data was obtained from the electronic medical record. Fisher’s exact was used for analysis. **Results:** One hundred and seventy-nine children had 237 infection episodes during the study period. Eighty-one patients (45%) were male and the median age was 5.3 years. The most prevalent MDRGNs included: Escherichia coli (154, 65%), Klebsiella spp (52, 22%), and Enterobacter spp (16, 7%). Escherichia coli was significantly more likely than other pathogens to be isolated from the urine (P = 0.008). Compared to multi-drug resistant E. coli, patients with a non-E. coli MDRGN were significantly more likely to have an underlying medical condition, recent hospitalization and antibiotic use (P≤0.001 for each, Table 1). A carbapenem was administered in 32% (75/237) of infection episodes. There were only 6 carbapenem resistant organisms. **Conclusions:** In our study, E. coli was the most frequent MDRGN. Most patients with a non-E. coli MDRGN infection episode had an underlying medical condition, recent hospitalization and antibiotic use. Carbapenem resistance was infrequent, though surveillance studies are needed to identify changing antibiotic resistance patterns and to direct prevention measures.

**Figure:**